# Air Quality Dispersion Modelling to Evaluate CIPP Installation Styrene Emissions

**DOI:** 10.3390/ijerph192113800

**Published:** 2022-10-24

**Authors:** Elizabeth Matthews, John Kraft, Gazi Hossain, Anthony Bednar, Charles Laber, Shaurav Alam, Tanvir Manzur, John Matthews, Jason Howell, Sven Eklund

**Affiliations:** 1 Trenchless Technology Center, College of Engineering and Science, Louisiana Tech University, Ruston, LA 71272, USA; 2 Engineering Research and Development Center, United States Army Corps of Engineers, Vicksburg, MS 39180, USA; 3 Department of Civil Engineering, Bangladesh University of Engineering and Technology, Dhaka 1000, Bangladesh; 4 Faculty of Chemistry, College of Engineering and Science, Louisiana Tech University, Ruston, LA 71272, USA

**Keywords:** CIPP resin, pipeline rehabilitation, styrene, AERMOD, public safety, trenchless technology

## Abstract

Cured-in-place pipe (CIPP) is one of the most popular in situ rehabilitation techniques to repair sewer and water pipes. While there are multiple approaches to curing CIPP, steam-curing of styrene-based resins has been found to be associated with air-borne chemical emissions. Health officials, utilities and industry representatives have recognized the need to know more about these emissions, especially styrene. Such concern has led to multiple studies investigating the concentrations of volatile organic compounds on CIPP installation sites. This study expands upon previous effort by modeling worst-case, steam-cured CIPP emissions over a 5-year weather record. The effort also includes calibration of the model to emissions averages over the work day rather than instantaneous field measurements. Dispersion modelling software, AERMOD, was utilized to model the styrene component of CIPP emissions on two CIPP installation sites in the US. Based on the analysis results, it was found that the styrene emitted from stacks dissipates rapidly with styrene concentrations only exceeding minimum health and safety threshold levels at distances close to the stack (2 m or less). The values predicted by the model analysis are comparable with the field measured styrene concentrations from other studies. Current safety guidelines in the US recommend a 4.6-m (15-ft) safety perimeter for stack emission points. The results of this study indicate that significant and lasting health impacts are unlikely outside recommended safety perimeter. The results also validate the importance of enforcing recommended safety guidance on steam-cured CIPP sites.

## 1. Introduction

The US infrastructure obtained an overall grading of “C-” in the 2021 report card published by the American Society of Civil Engineers (ASCE). The report shows that the grade score of infrastructures for drinking water, storm water, and waste-water categories were equal or even lower, “C-”, “D” and “D+”, respectively [1]. The deteriorating conditions of the pipe networks associated with such poorly ranked infrastructures have led to the increased need for fixing this issue. Since millions of feet of buried pipes are overlapping each other at their underground locations especially in urban areas, open cut replacement of deteriorated buried pipe network is often not a viable restoration option due to its high socio-economic impacts. Therefore, trenchless rehabilitation of buried pipe network has been showing increasing demand and continuous growth as it can avoid socio-economic impacts on communities by not reducing traffic flow, causing business losses, etc. [2,3].

One of the most popular and practiced trenchless rehabilitation techniques is cured-in-place pipe (CIPP) because the overall installation process trigger minimal disturbance to the communities. As a result, the application of CIPP has seen significant growth world-wide in the last 30 years and accounted for the majority of all pipe rehabilitation currently in practice [4]. The CIPP method involves installing a resin-impregnated liner cured on the interior walls to retrofit the existing deteriorated pipes. The hardening of the resin impregnated liner is ensured through different curing methods (steam, hot water, or ultra-violet light) among which steam curing is utilized most often. When liners are steam-cured, an exhaust is produced during the curing process that is vented into the surrounding environment typically via a short stack (1.85 m or less). As CIPP installation has become more common, health officials, utilities, and industry representatives have recognized the need to know more about the air-borne chemicals emitting through the short stack. Evaluations of steam-cure emissions done in previous studies have found that these emissions contained a variety of volatile organic compounds (VOC) [5,6,7,8].

In the case of CIPP liners that are impregnated with styrene-based thermoset resins, styrene has been identified as a hazardous VOC of concern and multiple studies have capture concentrations exceeding recommended exposure limits [6,8]. However, these studies primarily focused the exhaust manhole, plume, and rogue emissions where maximum concentrations would be expected [6,8]. There have also been other studies measuring VOC concentrations in air emissions during CIPP installation, but the studies did not directly measure styrene [7]. As per U.S. Department of Health and Human Services (HHS), breathing high levels of styrene has negative impacts on the nervous system [9] with impacts affecting the central, peripheral, and autonomic nervous systems reported in the literature [10,11,12,13,14]. Therefore, workers have potential to be harmed from exposure to a higher than recommended level of styrene. Prior to [5], only one study collected styrene emission concentrations in the surrounding environment. This study, completed by the Institute for Underground Infrastructure (IKT) in Germany, found that styrene emission concentrations at 5 m or greater were relatively low with the highest reading at 20 ppm [15].

The study done by [5] addressed the gap in previous studies by directly capturing styrene concentrated air in sampling canisters and sorbent tubes at both source points and surrounding areas to fully evaluate the risk to workers and communities. The study collected data from multiple sites across various geographies and site characteristics and found two locations on CIPP installation sites where potential styrene exposure could result in negative health impacts. These two locations include the liner truck, where CIPP liners are stored and transported prior to installation, and areas within 3 m (10 feet) of the exhaust stack [5,16].

While more recent effort has captured a more complete picture of styrene exposure risk to workers on CIPP sites, most of the previous evaluations have relied on field captured concentration data and have largely ignored utilizing air dispersion modelling in evaluation of risk. The advantage of utilizing air dispersion modelling is the abilities to evaluate risk of a range of weather conditions as weather can have a significant impact on dispersion of VOCs. Therefore, collecting data onsite during a particular period only represents, at best, the potential field concentrations associated with a limited range of potential weather variables. While [5] addressed the application of air quality dispersion modelling software to model CIPP emissions at multiple sites, the work focused on comparing modeled data to instantaneous field measurements and considered only one year of weather data.

In order to better evaluate potential risk to human health from CIPP steam-cure emissions considering a variety of environmental variables, this study expands upon [5] by utilizing AERMOD to model the styrene component of CIPP emissions for two of the six geographical sites modeled in the original study and by expanding the analysis to five years of weather data. This study also utilizes a model calibration approach based on time-weighted average field samples. The study showed that styrene concentration levels exceed minimum health and safety threshold limits near the emission stack, yet the recorded levels dropped as the distance from the emissions points increased. The measurements in [5] indicated that styrene exposure leading to significant or lasting health impacts are likely to occur outside the currently recommended safety perimeter. For this study, the AERMOD modelling system is utilized to estimate styrene concentrations for two sites at multiple receptor points. The primary reason for modelling emissions was to evaluate the emissions concentrations for possible worst case scenario” over a range of weather conditions to evaluate human health impacts in the nearby environment. The modeling is also used to evaluate whether the currently recommended 4.6-m (15-ft) safety perimeter in the US [17] around the stack location is sufficient to protect workers and the surrounding communities.

## 2. Materials and Methods

### 2.1. Study Locations

The first study area location is the City of Shreveport, which lies in the northwest corner of the state of Louisiana in the United States. With a population of approximately 200,000 in 123 square miles, Shreveport is the third largest city in Louisiana. The second location is the City of Aurora, which is near Denver in the state of Colorado in the United States. Aurora has a population of approximately 325,000 and is just over 150 square miles.

**Figure 1:**
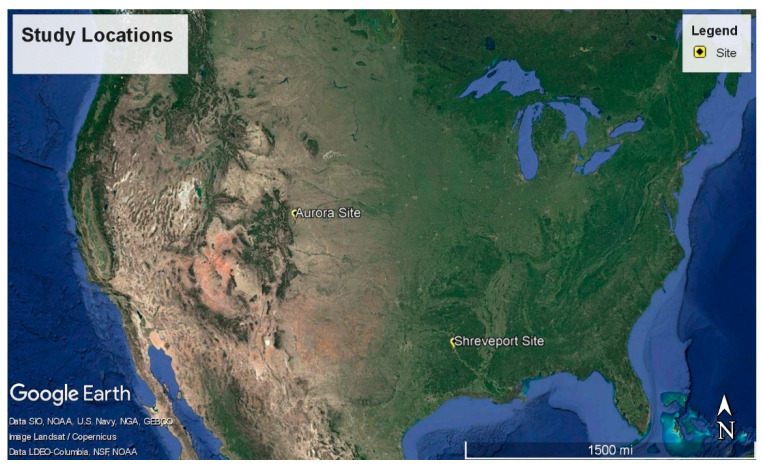
Location of Study on the Continental USA Scale.

Selection of these two locations were primarily governed by their variation in climate and elevation, proximity of mountains and flat lands, since both climate and geographic elevation play a significant role in the dispersion of air emissions. In addition to the variation in climate, these two sites also had variation in the pipe configuration (i.e., pipe diameter and length). Table 1 summarizes some of the site characteristics for these two locations, including the land use, climate, and elevation. The table also includes pertinent details about the pipes undergoing relining on both CIPP jobsites. Figure 1 shows the google earth locations of each site within the continental U.S.

### 2.2. AERMOD Modelling System

AERMOD is the EPA-preferred regulatory software developed for modelling air dispersion. The software utilizes a steady-state Gaussian algorithm for modelling air dispersion for distances less than 50 km from a stationary source such as a stack [18,19]. The model considers meteorological data, terrain data, and building data to determine plume behavior and calculates concentrations for pollutants of interest. The interaction of the plume with buildings can also cause downwash effects, where pollution can be directed down toward the ground surface rather than up into the atmosphere [20]. While AERMOD has had limited application to analyzing construction site emissions [21,22], other studies have utilized AERMOD to model to evaluate accidental pollutant release [23], nitrogen dioxide (NO_2_) release from cement plants [24], emissions from agricultural facilities [25], roadways [26], and a variety of industrial facilities [27,28,29].

**Figure 2:**
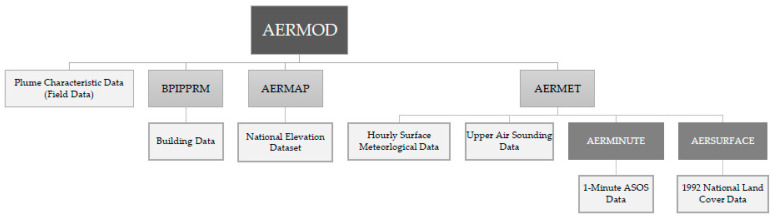
AERMOD System and Data.

AERMOD consists of a system of modules, which include pre-processors AERMET, AERMAP, AERSURFACE, and BPIPPRIM [19,30,31]. Figure 2 outlines the entire AERMOD system including all components and data. AERMOD calculates the pollution concentrations for a set of analysis point locations based on all the input data provided to the model. A receptor grid defines the set of analysis points where pollutant concentrations are generated in the AERMOD output file. In addition to the AERMOD system, AERPLOT can be used to convert the final output data from AERMOD for visualization in Google Earth [31]. AERMET is used to process meteorological data to create the atmospheric boundary layer parameters needed to calculate dispersion concentrations in AERMOD. Data utilized by AERMET include hourly surface meteorological data and upper air sounding data from the nearest station to each site. Typically, these stations are located at airports. Automatic Surface Observing Systems (ASOS) 1-min wind data is also used and is processed in AERMINUTE to create the hourly average winds for input into AERMET [30]. AERSURFACE, which processes land surface characteristics for AERMET, utilizes land cover surface data [31]. AERMAP processes terrain data to develop the elevation and hill-height scaling factors for the receptors, where the dispersion concentrations are calculated in AERMOD. BPIPPRIM is used for calculating downwash values base on the surrounding building characteristics [31] for input into AERMOD.

There are some limitations to the AERMOD modeling system and its related model support programs that affected the study. First, the averaging period options are limited to 1, 2, 3, 4, 6, 8, 12, or 24 h. The model is also designed to output results in µg/m^3^, which makes it difficult to convert the model results to ppm for ambient conditions outside of standard temperature and pressure [32]. The low quality of the AERPLOT graphical output into Google Earth makes it difficult to interpret results and incorporate into technical documents. Additionally, when accounting for downwash affects, sloped roofs could not be modeled in BPIPPRM.

### 2.3. Model Parameters and Setup

In order to setup the AERMOD dispersion model, source plume characteristics were measured on each site that was considered. The parameters needed to model each site included the stack plume velocity (m/s), stack height (meters), stack diameter (meters), stack temperature (K) and emission rate for the pollutant of interest (g/s). Plume velocity, stack height, stack diameter, and temperature were measured directly with instruments in the field. Flow velocity and temperature of the steam plume from the curing liner were measured using a TSI VelociCALC anemometer and Venier temperature probe, respectively. Multiple stack temperature measurements were taken during the curing process and the stack temperature for the purpose of modelling was taken to be the average plume temperature of all temperature measurements collected in the field. Plume velocity was measured at the estimated peak temperature during curing, although the velocity was observed to vary over the curing process.

**Figure 3:**
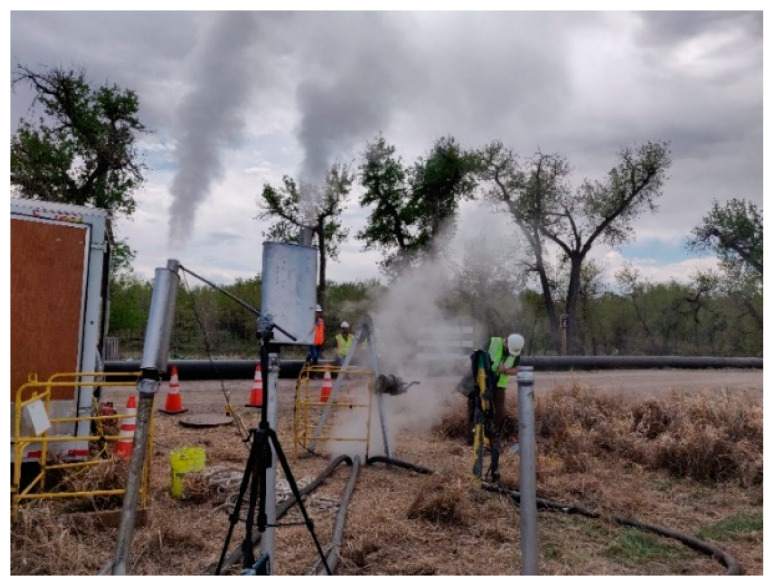
Stack Configuration at Aurora Site.

Emission concentrations at both the plume and surrounding area were determined through sampling followed by laboratory gas chromatography/mass spectrometry (GC/MS) analysis. While both sampling canisters and sorbent tubes were collected on site, the sorbent tube data was primarily used to support the modelling effort that is the focus of this study. Multiple sorbent tubes were attached to various stationary locations on each site. It should be noted that while both sites were modeled as a single stationary stack, the CIPP installation site in Aurora had three stacks (Figure 3).

While other compounds could have been modeled, based on the laboratory GC/MS data, only styrene demonstrated measured concentrations in the field that had the potential to pose a health hazard. It was decided based on these measurements that the modelling effort would focus only on styrene. The styrene emission rate was extrapolated from the GC/MS results for canisters collected directly at the steam plume. Concentration results for styrene given in the laboratory analysis results were converted from parts-per-million to grams-per-cubic-meter using the ideal gas law at standard conditions, according to Equation (1). The emission rate was then calculated utilizing Equation (2).

(1) $$\mathrm{Concentration}\;\left(\frac{g}{m^{3}}\right)=\frac{\mathrm{Concentration}\;\left(\mathrm{PPM}\right)\times 101.32\times 104.15}{8.3144\times 273.15\times 1000}$$

where,

101.32 is the standard pressure (kPa)
273.15 is the standard temperature K
104.15 is the molecular weight of styrene, $\frac{g}{\mathrm{mol}}$
8.3144 is the gas constant, $\frac{m^{3}\times \mathrm{Pa}}{K\times \mathrm{mol}}$

(2) $$\mathrm{Emission}\;\mathrm{Rate}\;\left(\frac{g}{s}\right)=\mathrm{Concentration}\;\left(\frac{g}{m^{3}}\right)\times \frac{\pi }{4}\times D_{\mathrm{stack}}^{2}\times V_{\mathrm{stack}}$$

where, $D_{stack}$ is the stack diameter in meter and $V_{stack}$ is the stack velocity in $\frac{m}{s}$.

The stack X- and Y-coordinates were determined based on Universal Transverse Mercator system. The stack X- and Y-coordinates were either measured in the field using the Global Positioning System (GPS) receivers on a cellular phone or through estimating the location based on geographic information system (GIS) or Google Earth imagery. The stack elevation (z-coordinate) was determined based on Light Detection and Ranging (LIDAR) data available through online sources [33] and/or [34].

In order to model downwash effect, building data was collected for input into the BPIPPRM pre-processor. Of the two sites, only the Shreveport site consisted of nearby residential structures. The Aurora site was located in a rural field. For modelling the residential building environment in Shreveport, building corner X, Y coordinate, building elevation and building story height data was collected from a variety of sources. Building X, Y coordinates were derived from Google Earth. The elevation of each building was derived from LIDAR [33] data. The building height for each story was estimated based on Google Street View imagery. For residential structures, the sloped roofs were modeled as flat roofs as AERMOD does not have a way to consider slopped roof. These data were used within the input files for the BPIPPRM preprocessor to determine which buildings could have/had downwash effects.

For the AERMINUTE preprocessor, ASOS data was extracted from National Oceanic and Atmospheric Administration (NOAA) online data sets [35]. For AERMET, hourly surface meteorological and upper air sounding data were extracted from NOAA online data sources [35,36] and used for the model. For AERSURFACE, the 1992 National Land Cover Database Dataset was obtained from the Multi-Resolution Land Characteristics (MRLC) Consortium [37]. For AERMAP, digital terrain data was required, which consisted of the 1/3 rd arc-second digital elevation model National Elevation Dataset (NED) data. This NED data was collected from the United States Geological Survey (USGS) [38].

For AERMOD concentration calculations, the receptor grid at each site was set as polar grid with 36 total points (10-degree intervals) at each of the defined distances (2, 3, 4, 6, 8, 15, 20 and 45 m). The origin or center of the polar grid was set as the main stack location for each site. The distances were selected to match as close as possible the distances of the surrounding area sorbent tube field measurements relative to the stack location.

### 2.4. Model Calibration

While it would have been more accurate to utilize a conversion equation accounting for local ambient conditions, the calculated emission rates resulting from Equations (1) and (2) were utilized as a starting point for calibrating the models for both sites. Some of the input parameters were adjusted so that the model simulation better matched the field data. The authors based their calculated emission rate off of one sample, which was taken at or near peak curing temperature. This represents one data point in time, but in reality the emission rate does vary over the entire curing process and would also vary during the 8 to 10-h workday window. Input parameters were adjusted during calibration so that the model results matched sorbent tube concentrations collected at stationary points in the field. At the Aurora site, duplicate samples were collected at each location. For the purpose of the modelling effort, duplicate results at individual locations were averaged.

In order to calibrate the model to the sorbent tube concentrations, parameters were adjusted include the velocity, stack height and emission rate. Only the 24-h weather data that aligned with the dates of the field visits were used during calibration. Table 2 summarizes the model stack parameters both measured and adjusted. Since the model was being calibrated to the sorbent tube measurements, the flagpole height was set in the AERMOD input files to match the mounted sorbent tube height, which was 0.45 m (1.5 feet). The averaging time for the model was set to match as close as possible to the sample time for the sorbent tube since the emission concentration in the samples are averaged over the entire sample time. For Shreveport, the sample time was 10 h, and the model averaging time was taken to be 8 h. The AERMOD model does not allow for an averaging time to be set to 10 h. Therefore, the model averaging period was conservatively set to 8 h. For Aurora, the sample time was 7.8 h, and the model averaging time was taken to be 8 h.

**Figure 4:**
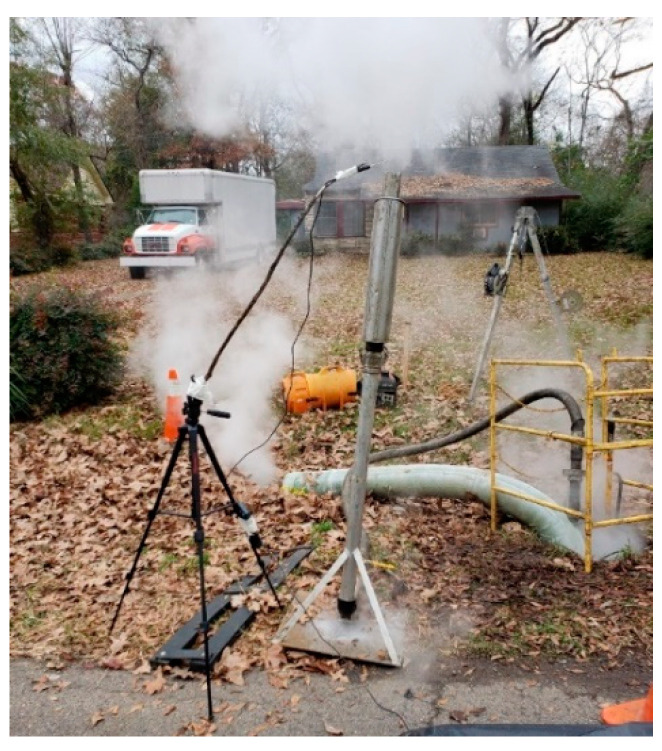
Emissions at Shreveport Site.

There were some field conditions including rogue emissions that were difficult to capture within the model as point sources. Ground level rogue emissions are common at the location of the termination manhole and liner end on CIPP installation sites (see Figure 4). Since these emissions would be captured by the sorbent tubes, it was not expected that the model would match the field data exactly. Effort was made to match the calibration model runs to field data closest to the stack location to account for some of the impacts of the ground level emissions at each site.

### 2.5. Model Simulations& Health Impacts

Once the model was calibrated, the model was analyzed across all hourly meteorological data for a period of five years (January 2016–December 2020) for both sites. An averaging period of 1-h with output concentration representing the highest concentrations was simulated to capture the worst-case-scenario for health hazard. The concentrations were geographically compared to the suggested 4.6-m (15 ft) safety perimeter [17]. Concentrations within and outside the perimeter were compared to published regulatory guidelines from the Environmental Protection Agency (EPA). Acute Exposure Guideline Levels (AEGL), as established by the EPA for hazardous substances, represent threshold exposure limits for the general public [39]. AEGL exposure limits specific to styrene and the corresponding health impacts associated with each AEGL (AEGL- 1 to 3) summarized in Table 3.

## 3. Results

### 3.1. Model Calibration

**Figure 5:**
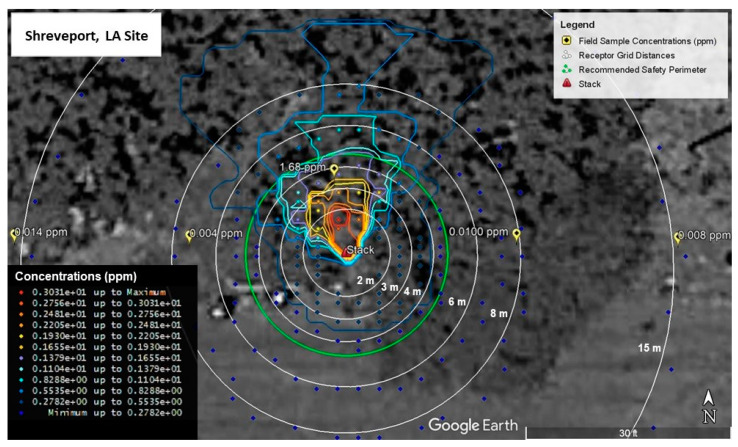
Modelled Styrene Emission Calibrated to Field Sample Concentrations (Shreveport, LA, USA).

**Figure 6:**
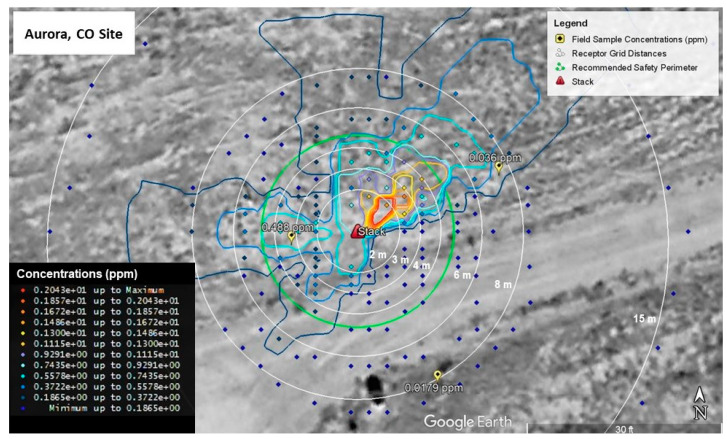
Modelled Styrene Emission Calibrated to Field Sample Concentrations (Aurora, CO, USA).

Calibration model runs for Shreveport, LA and Aurora, CO are shown in Figure 5 and Figure 6, respectively. As can be seen in Figure 5, the modeled emission concentrations closest to the field measured concentration of 1.68 ppm fall into a range from 1.38 to 1.93 ppm. This was the closest sorbent tube field measurement to the stack, so the modeled values were calibrated to more closely match this value. Measurements farther from the stack all measured at or below 0.01 ppm. Modeled values around the same distance from the stack as measured values are at least below 0.3 ppm with values closest to the field measurements falling within a range between 0.03 and 0.08 ppm.

For the Aurora site (Figure 6), the upwind measured concentration of 0.488 ppm is near modeled concentration values ranging from 0.372 ppm to 0.744 ppm. Similar to the Shreveport site, the model was calibrated to more closely match this value since it was nearest the stack. The downwind value of 0.036 ppm is located near modeled values ranging from 0.186 to 0.372 ppm, while the crosswind value is located in a field of modelled values less than or equal to 0.186 ppm. Values nearest the crosswind measurement are a better match to the field measurement with the nearest modelled values being between 0.007 and 0.012 ppm.

### 3.2. Model Simulations& Health Impacts

**Figure 7:**
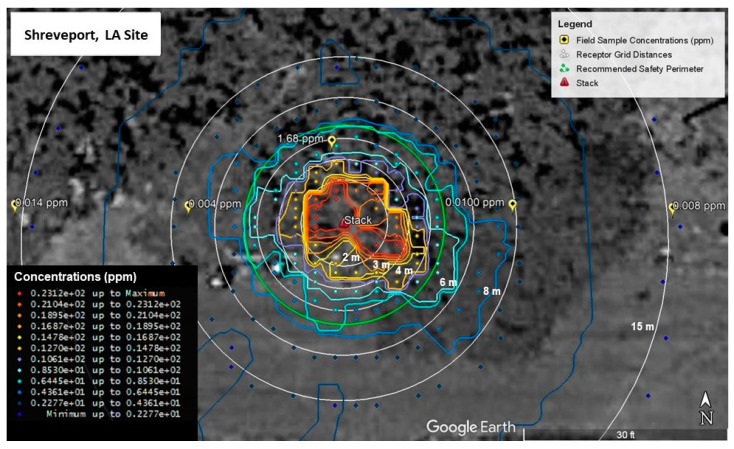
5-year Modelled Styrene Emissions (Shreveport Site).

**Figure 8:**
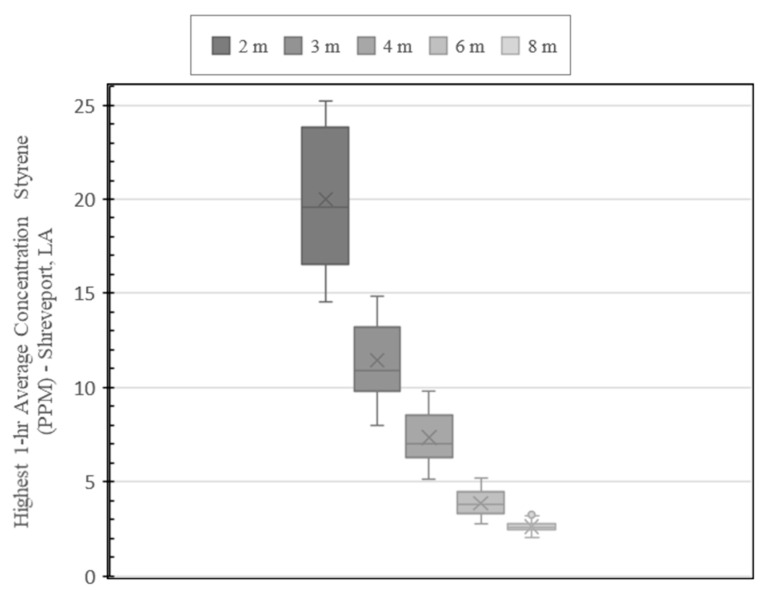
5-year Modeled Styrene Concentrations by Radial Distance (Shreveport Site).

AERMOD model run results for the Shreveport site are shown in Figure 7 and Figure 8. Figure 7 shows the map of the highest 1-hr average concentrations modeled in AERMOD over the 5-year weather period. The map also includes the recommended 4.57-m (15-ft) safety perimeter. Concentrations higher than 12.7 ppm, represented by warm colors, are contained within the perimeter. To better visualize the modeled concentration with respect to distance, the modeled values are depicted by the box-and-whisker plot shown in Figure 8. The highest modelled concentrations occur within 2 m of the stack and range from 14.5 to 25.2 ppm. Between 2- and 3-m, concentrations range between 8.01 and 14.8 ppm. The modelled concentrations between 3 and 4 m ranged from 5.11 to 9.79 ppm.

Comparing the modeled concentrations to the Environmental Protection Agency’s Acute Exposure Guideline Levels (AEGL), only the AEGL-1 exposure limit of 20 ppm is exceeded, and this only occurs within the first 2 m.

**Figure 9:**
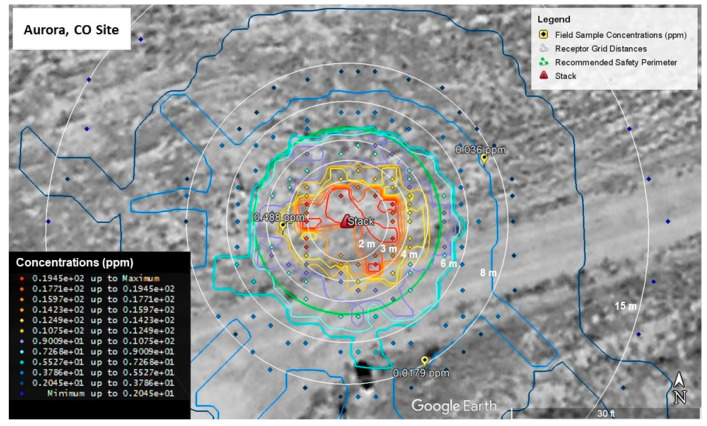
5-year Modelled Styrene Emissions (Aurora Site).

**Figure 10:**
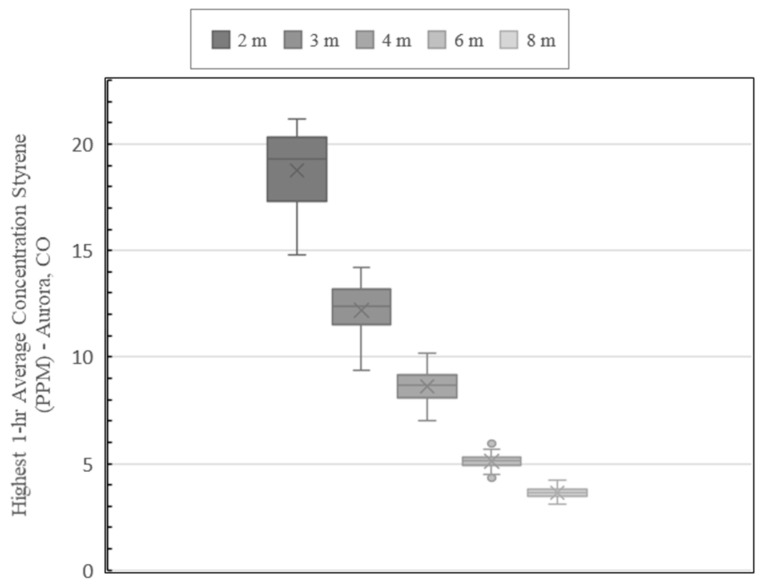
5-year Modelled Styrene Concentration by Radial Distance (Aurora Site).

Figure 9 and Figure 10 show the AERMOD model run results for the Aurora site. Figure 9 shows the map of the highest 1-hr average concentrations including the recommended safety perimeter. Like the plot developed for the Shreveport site, concentrations represented by warm colors are contained within the perimeter which represent concentrations 10.7 ppm and higher. The box-and-whisker plot of the Aurora site concentrations (Figure 10), show a similar pattern to the results obtained for the Shreveport site. The highest modelled concentrations occur within 2 m of the stack and range from 14.8 to 21.2 ppm. Between 2 and 3 m, concentrations range from 9.35 to 14.2 ppm. The concentrations ranged from 7.04 to 10.2 ppm between 3 and 4 m. Like the Shreveport site, the AEGL-1 exposure limit of 20 ppm is exceeded only within the first 2 m.

## 4. Discussion

Overall, even though both sites represent differing climates, elevations and pipe configurations, the results are similar for both the locations. Within 2 m, the Shreveport site has a slightly higher mean concentration of 20 ppm compare to the Aurora site at 18.8 ppm. Exposure to 20 ppm at any duration, represented as AEGL-1, generally results in discomfort that is transient, reversible and not disabling [9]. This data modeling suggests, in general, that the styrene dissipates rapidly and that significant and lasting health impacts are not expected outside of 2 m.

These values are also comparable to instantaneous styrene concentration evaluations around CIPP installation stacks presented in other studies. For example, [5] measured concentrations across 9 sites ranging from 19.5 to 36.1 ppm at 1.83 m (6 ft) or less from the stack. Outside of 3.05 m (10 ft), none of the measurements taken exceeded AEGL-1 limit. Another study (IKT) measured styrene concentrations at various distances from a CIPP exhaust point and recorded the highest concentration of 20 ppm at 5 m. Measurements beyond 5 m were all 11 ppm or less [15]. Although the model did not show any concentrations outside of 2 m exceeding 20 ppm, both of these studies reported styrene concentrations measured directly in the field that are, for the most part, within the range the model predicts.

For both sites, the results suggest that the currently recommended safety perimeter of 4.6 m (15 ft) around the stack is sufficient to protect workers and community members. The IKT study recommended a safety perimeter of 5 m (16-ft), which helps to validate both the model results and the current guidance in the US [15]. Compared to the one-year AERMOD modelling study results completed previously [5], the concentration results calculated in the five-year model period were higher. It is important to note that the models run in the one-year study were over a 1-h averaging period and were calibrated to instantaneous field measurements rather than styrene emissions collected over the workday (i.e., sorbent tubes) [5]. It is difficult to directly compare the results of these two studies since the model approaches were different. However, both studies indicate that exposure impacts are low beyond the recommended safety perimeter.

## 5. Conclusions

This study presents the results of an AERMOD dispersion model analysis of styrene emissions from the installation of steam-cured CIPP liner, which is one of the most popular in situ rehabilitation techniques for sanitary sewer, storm sewer, and drinking water pipes. Two site installations, with diverse topography located in Shreveport, LA and Aurora, CO, in the US were analyzed. These two sites represent differing climates, elevations, and pipe configurations. The dispersion analysis incorporated 5-years of weather data and was utilized to simulate the highest 1-h average concentrations at various distances from an emission stack location. The following conclusions and recommendations were drawn from the results of the study:

1. Styrene dissipates rapidly with concentrations only exceeding minimum health and safety threshold levels (AEGL-1) at distances close to the emissions stack (2 m or less).
2. Significant and lasting health impacts are unlikely to occur outside of the currently recommended 4.6-m (15-ft) safety perimeter for stack emission points on CIPP sites.
3. Styrene concentration values predicted by the model for both sites are comparable to field measured styrene concentrations from other studies. The modeled values of 18.8 ppm and 20 ppm fall within a range of instantaneous field measure values reported in previous literature

The results of this study suggest that the recommended safety perimeter would aide in protecting workers while also validating the need of utilizing the perimeter to protect against negative health impacts associated with minimum safety threshold levels. The impact of the results of this study help to further clarify the need for continued implementation of safety recommendations on steam-cured CIPP sites. Recommendations for future study include modeling other styrene emissions associated with CIPP such as volatilization of styrene from uncured liners in storage, rouge emissions, and stack emissions associated with specific phases of the curing process.

One limitation of the study is difficulties accounting for ambient conditions in the conversion of concentration values within the model itself. Since a wide range of historical weather conditions are modeled over the five-year period, accounting for ambient conditions for all calculated concentrations would have been difficult. The conversion factor embedded in the model coding utilizes the standard temperature and pressure conversion for converting the output to parts-per-million. The model was based on field data collected in a previous study, which was more comprehensive than previous work but included a limited number of data points. Collection of more field data at an expanded number of locations would help to further validate the model. Additional collection of emission data at the stack location, would also help to capture variation in the stack emission rate over the CIPP installation process.

## Figures and Tables

**Table 1:** Site Location and Pipe Characteristics for Shreveport, LA and Aurora, CO, USA.

City	Location Latitude Longitude	Land Use	Climate	Elevation m (ft) above MSL	Pipe Diameter mm (in)	Pipe Length m (ft)
Shreveport, LA	N 32°29′23.41 W 93°47′57.65″	Residential	Humid & Wet	61 (200)	203 (8)	185.4 (608)
Aurora, CO	N 39°44′54.93″ W 104°47′53.33″	Rural	Dry	1646 (5400)	914 (36)	106.1 (348)

**Table 2:** Measured and Adjust Model Stack Parameters.

Site (Site Visit Date)	Model Stack Parameters						
	Velocity (m/s)	Height (m)	Diameter (m)	AVG Temp (K)	Styrene Concentration (ppm)	Calculated Emission Rate (g/s)	Adjusted Emission Rate (g/s)
Shreveport (12 December 2018)	21.23 * 25	1.78 * 0.5	0.056	287.5	0.0500	0.00001	* 0.15
Aurora (14 May 2019)	23.5	1.8	0.05	298.9	25.4	0.00621	* 0.18

* Adjusted parameter values utilized in models.

**Table 3:** Summary of AEGL Styrene Exposure Limits and Health Impacts.

AEGL Styrene Exposure Limit	Impact on Health
Level-1 ○ Styrene Exposure Limit: 20 ppm (for any duration)	General population, including susceptible individuals, could experience notable discomfort, irritation or certain asymptomatic, non-sensory effects Effects are not disabling and are transient and reversible upon cessation of exposure
Level-2 ○ Styrene Exposure Limit: 230 ppm for exposure <10 min ○ Styrene Exposure Limit: 160 ppm for exposures from 10 to 30 min ○ Styrene Exposure Limit: 130 ppm for exposures >30 min	General population, including susceptible individuals, could experience irreversible or other serious, long-lasting adverse health effects or an impaired ability to escape.
Level-3 ○ Styrene Exposure Limit: 1900 ppm for exposures <30 min ○ Styrene Exposure Limit: 1100 ppm for exposures from 30 min to 1 h ○ Styrene Exposure Limit: 340 ppm for exposures >1 h	General population, including susceptible individuals, could experience life-threatening health effects or death.

## Data Availability

Some of the data, models, or code generated or used during the study are available in a repository online in accordance with funder data retention policies. The data in the study referenced in the text as the NASSCO Phase II study [5] is available at online at https://www.nassco.org/news/CIPP-study (accessed on 10 August 2022). Additional data not captured within the NASSCO Phase II study source can be provided upon request by contacting the corresponding author.

## Abbreviations

AEGL	Acute Exposure Guideline Level
ASCE	American Society of Civil Engineers
ASOS	Automatic Surface Observing Systems
CDC	Centers for Disease Control
CIPP	Cured-In-Place Pipe
EPA	US Environmental Protection Agency
GC/MS	Gas Chromatography/Mass Spectrometry
GIS	Geographic Information System
GPS	Global Positioning System
HHS	US Department of Health and Human Services
IKT	Institute for Underground Infrastructure
LIDAR	Light Detection and Ranging
MRLC	Multi-Resolution Land Characteristics
NASSCO	National Association of Sewer Service Companies
NED	National Elevation Dataset
NOAA	US National Oceanic and Atmospheric Administration
PPM	Parts Per Million
USGS	United States Geological Survey
VOC	Volatile Organic Compound
